# Successful Conservative Management of Acute Microperforation in a Diverticulum Within a Meckel’s Diverticulum: A Case Report

**DOI:** 10.7759/cureus.72834

**Published:** 2024-11-01

**Authors:** Hiraku Sedogawa, Satoshi Yoshikawa, Takeshi Ueda

**Affiliations:** 1 Emergency and General Internal Medicine, Rakuwakai Marutamachi Hospital, Kyoto, JPN

**Keywords:** conservative management, diverticulitis, diverticulum, laparoscopic resection, meckel's diverticulum, micro-perforation

## Abstract

Meckel's diverticulum is a common congenital gastrointestinal anomaly that can lead to various complications. This case report presents a rare instance of microperforation of a diverticulum within Meckel's diverticulum. A 31-year-old male presented with a two-day history of progressively worsening, intermittent right lower quadrant pain and fever. The patient reported dull pain that migrated from the epigastric region to the right lower quadrant over the course of two days. The pain was exacerbated by movement. Blood tests revealed WBC 13,900/μL and CRP 5.30 mg/dL. Given the patient's characteristic clinical presentation suggestive of appendicitis, an initial ultrasound examination was performed. However, the ultrasound failed to reveal findings typical of appendicitis. Considering the possibility of conditions mimicking appendicitis, a contrast-enhanced computed tomography (CT) scan was subsequently conducted. The CT scan revealed a blind-ending intestinal structure branching from the jejunum, surrounded by fat stranding and free air. These findings were suggestive of a microperforation of a diverticulum within Meckel's diverticulum. The patient was initially managed conservatively with bowel rest and antibiotic therapy, resulting in significant symptom improvement. Subsequent laparoscopic resection confirmed the presence of a diverticulum within Meckel's diverticulum. This case highlights the diversity of Meckel's diverticulum complications and demonstrates the potential for successful conservative management in select cases of microperforation of diverticulum within a Meckel’s diverticulum.

## Introduction

Meckel's diverticulum is a congenital true diverticulum originating from the persistent omphalomesenteric duct, occurring in approximately 2% of the general population [1]. As a true diverticulum containing all three layers of the intestinal wall, Meckel's diverticulum is often asymptomatic but can occasionally lead to complications such as bleeding or intussusception. Diverticulitis is a relatively common complication of Meckel's diverticulum, presenting with symptoms similar to acute appendicitis [2]. However, cases of diverticulitis within Meckel's diverticulum are relatively rare, and the clinical presentation and optimal management strategies are not well established [3]. Particularly, no case report shows perforation of the diverticulum within Meckel's diverticulum, requiring special attention in diagnosis and treatment. This pathology may follow a different clinical course than typical Meckel's diverticulitis. Through this case, we aim to describe the clinical presentation, diagnostic process, and treatment strategy for this unique condition, providing valuable information for managing similar cases. Additionally, we seek to highlight the diversity of Meckel's diverticulum complications and emphasize the importance of diagnostic approaches for rare pathologies.

## Case presentation

A 31-year-old male with a history of hypertension and intussusception presented to the emergency department with right lower quadrant pain and fever. The patient had a history of intussusception in early childhood; however, the specific etiology could not be determined due to the unavailability of medical records and the patient's inability to recall details. According to the patient's recollection, the condition was managed conservatively, resulting in resolution without surgical intervention. Following this successful conservative management, no further follow-up was conducted. The pain had initially appeared in the epigastrium two days prior, migrating to the right lower quadrant on the day of admission. The pain was described as dull without radiation, exacerbated by eating and moving. The patient reported nausea for two days and recent fatigue, prompting him to check his temperature, which was 37.3°C. Over-the-counter analgesics provided no relief, and at this point, no antibiotics had been administered. He reported occasional alcohol consumption and denies illicit drug use and recent consumption of raw meat.

On physical examination, the patient's vital signs were notable for a low-grade fever of 37.7°C, tachycardia with a heart rate of 116 beats per minute, and slightly elevated blood pressure at 144/93 mmHg. His respiratory rate was 18 breaths per minute, and oxygen saturation was 100% on room air. The patient exhibited a shuffling gait due to abdominal discomfort. Cardiovascular and respiratory examinations were unremarkable, with normal heart sounds, and clear breath sounds bilaterally. Abdominal examination revealed a flat and soft abdomen. The patient reported significant spontaneous pain in the right lower quadrant, rating it as 7 out of 10 on the Numerical Rating Scale (NRS). There was marked tenderness to palpation in the same area, although no rebound tenderness was elicited. Both the heel drop test and tapping pain were positive, suggesting localized peritoneal irritation. The remainder of the physical examination was normal. Blood tests revealed WBC 13,900/μL and CRP 5.30 mg/dL. The renal and liver function tests were unremarkable. Initial clinical presentation of migratory abdominal pain and fever raised suspicion of acute appendicitis. Consequently, we performed an ultrasound examination as the first-line imaging modality. However, the ultrasound failed to visualize the appendix or reveal any findings suggestive of appendicitis.

**Figure 1:**
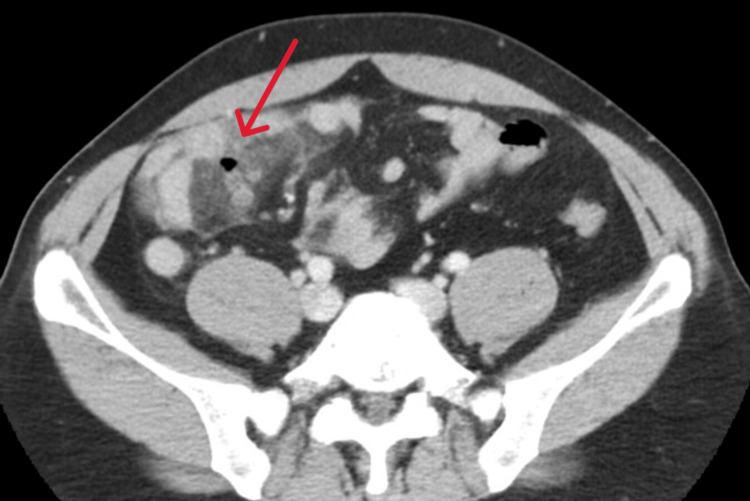
Intraabdominal free air and fat stranding around the Meckel's diverticulum (red arrow).

Given the inconclusive ultrasound results and the persistence of symptoms, we considered the possibility of other pathologies that could mimic appendicitis. To further investigate and differentiate potential causes, we proceeded with a contrast-enhanced CT scan. The scan showed no appendiceal enlargement or peri-appendiceal fat stranding. However, it revealed a blind-ending intestinal structure branching from the jejunum, surrounded by fat stranding. Free air was present within the fat stranding, but no abscess formation was observed (Figure 1).

**Figure 2:**
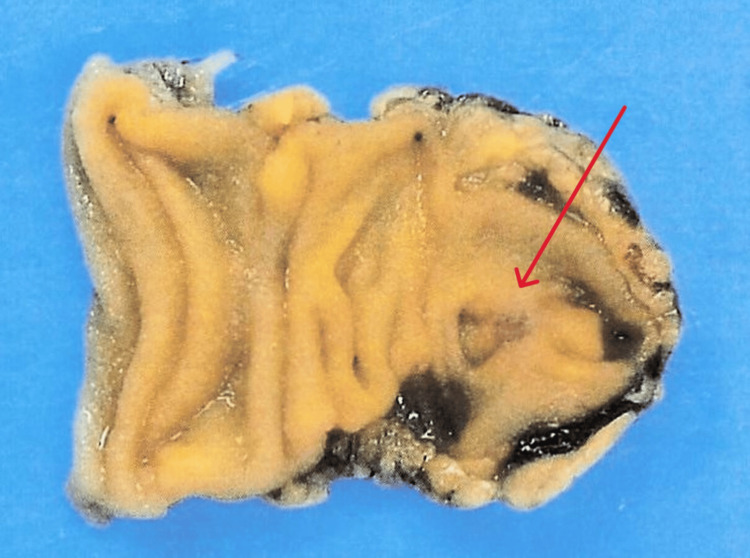
A pseudodiverticulum (red arrow) is observed within the resected Meckel's diverticulum.

On contrast-enhanced CT, the diverticula within the Meckel’s diverticulum were identified, suggesting microperforation of a diverticulum within the Meckel's diverticulum. After consultation with gastrointestinal surgery, conservative management with bowel rest and antibiotic therapy was initiated, given the localized nature of the inflammation. The patient was admitted for treatment with intravenous cefmetazole (CMZ) 6 g/day. By the third day of hospitalization, spontaneous pain improved to NRS 3/10, and blood tests showed improvement (WBC 6,000/μL, CRP 2.8 mg/dL). On the fifth day, pain resolved completely (NRS 0/10), allowing diet resumption without abdominal symptom exacerbation. CMZ was discontinued on the seventh day. Although surgical resection is typically recommended for Meckel's diverticulitis, the patient was informed about the lack of epidemiological data on the recurrence of microperforation of a diverticulum within Meckel's diverticulum. Referencing risk factors for asymptomatic Meckel's diverticulum [4], elective surgical resection was proposed for secondary prevention of complications. The patient consented to laparoscopic Meckel's diverticulum resection. The Meckel's diverticulum resection margins were set approximately 30 mm from the pseudodiverticulum, allowing for complete excision of the Meckel's diverticulum. The resected specimen confirmed the presence of a diverticulum within the Meckel's diverticulum (Figure 2), without evidence of perforation in the Meckel's diverticulum itself.

No ectopic pancreatic or gastric tissue was found. Upon surgical resection, the pseudodiverticulum was found to be sealed. Histological examination revealed no abnormalities in the Meckel's diverticulum, which is a true diverticulum. Therefore, it was concluded that the perforation had likely occurred in a diverticulum within the Meckel's diverticulum, which is classified as a pseudodiverticulum, rather than in the Meckel's diverticulum itself. The patient recovered without postoperative complications and was discharged five days after the surgery.

## Discussion

Meckel's diverticulum is the most common congenital gastrointestinal anomaly, present in 2% of the general population [1]. The cumulative incidence of complications in patients with Meckel's diverticulum ranges from 4.2% to 6.4% [5,6], including intussusception, volvulus, and diverticular bleeding [7]. Park et al. identified four criteria associated with an increased risk of symptom development: age (under 50 years), diverticulum length (≥2 cm), male gender, and presence of ectopic tissue [4]. This patient met three of these criteria, excluding ectopic tissue, indicating a 42% risk of becoming symptomatic. Various rare complications of Meckel's diverticulum have been reported, including perforated Meckel's diverticulum with carcinoid tumor [8], giant Meckel's diverticulum causing post-traumatic hematoma [9], Meckel's diverticulitis with intestinal malrotation [10], and axial torsion with gangrene [11]. These rare complications are often misdiagnosed, as exemplified by a case misdiagnosed as Crohn's disease [12]. This case also initially suspected as appendicitis revealed microperforation of the diverticulitis within Meckel's diverticulum, a first report to demonstrate the existence of these specific complications. While Meckel's diverticulitis and its perforation have been reported, often associated with enteroliths [13] or intradiverticular tumors [14], this case showed no evidence of these on CT or pathological examination. The diverticula within Meckel's diverticulum was a false diverticulum lacking a muscular layer, potentially increasing the risk of perforation similar to appendiceal diverticulitis [15]. This finding expands the diversity of Meckel's diverticulum complications and provides a new perspective on diagnostic and treatment approaches.

While many Meckel's diverticulum complications require acute surgical intervention [16,17], our case demonstrated successful conservative management under close monitoring, similar to the management of microperforation in appendicitis. This case suggests the possibility of non-surgical management in the acute phase for certain presentations of symptomatic Meckel's diverticulum, contrasting with the often-recommended surgical resection. This case highlights the importance of considering both common and rare complications of Meckel's diverticulum in the differential diagnosis of patients presenting with symptoms suggestive of acute appendicitis. While intestinal obstruction and hemorrhage are well-documented complications [1], the unique presentation of microperforation of the diverticulum within a Meckel's diverticulum in our case underscores the need for clinicians to maintain a high index of suspicion for atypical manifestations. It also highlights the necessity of appropriate imaging diagnostics and timely surgical intervention when needed. In this case, given the typical presentation of appendicitis, we initially performed an ultrasound to further increase the pre-test probability of appendicitis. However, the ultrasound findings did not reveal typical signs of appendicitis.

Consequently, we considered whether to proceed with either observation, CT imaging, or diagnostic laparoscopy. Each option presented its own drawbacks: CT imaging carries the risk of radiation exposure, while diagnostic laparoscopy poses potential perioperative complications, including anesthesia risks and allergic reactions to medications. Ultimately, after discussing the options with the patient, we opted for a CT scan, which successfully identified a microperforation of the diverticulum within Meckel's diverticulum. While CT imaging is not always necessary, we believe that following guidelines [18] and engaging in shared decision-making with patients can facilitate appropriate surgical interventions when indicated. Future accumulation and analysis of similar rare cases may lead to further improvements in diagnostic and treatment strategies for Meckel's diverticulum complications.

## Conclusions

Meckel's diverticulum, present in a small percentage of the population, can present with nonspecific abdominal symptoms similar to appendicitis. We report a case of microperforation originating from diverticulitis within a Meckel's diverticulum. There is no clear consensus on the management of diverticula within Meckel's diverticulum. When encountering perforated Meckel's diverticulitis, clinicians should consider not only enteroliths or intradiverticular tumors but also the possibility of perforated diverticulitis within the Meckel's diverticulum, potentially allowing for non-surgical management in the acute phase.
